# COVID-19 pandemic distress among a sample of Italian psycho-oncologists: risk of isolation and loneliness

**DOI:** 10.1177/0300891621992129

**Published:** 2021-02-15

**Authors:** Anna Costantini, Eva Mazzotti, Samantha Serpentini, Angela Piattelli, Dorella Scarponi, Gabriella De Benedetta, Marco Bellani

**Affiliations:** 1 Psycho-Oncology Departmental Unit, Sant’Andrea Hospital, Sapienza University of Rome, Rome, Italy; 2 Department of Clinical and Molecular Medicine, Sant’Andrea Hospital, Sapienza University of Rome, Rome, Italy; 3 Psycho-Oncology Unit, Veneto Institute of Oncology IOV-IRCCS Comprehensive Cancer Center, Padua, Italy; 4 UOC Oncologia–Dipartimento Oncoematologico, Azienda Ospedaliera Cosenza, Cosenza, Italy; 5 Paediatric Unit, Sant’Orsola Hospital, Bologna University, Bologna, Italy; 6 Hematology Department, National Cancer Institute, Foundation Pascale, Naples, Italy; 7 Psycho-oncology Unit, Department of Medicine and Surgery, University of Insubria, Varese, Italy

**Keywords:** COVID-19, CPDI, isolation, loneliness, peritraumatic distress, psycho-oncologist

## Abstract

**Purpose:**

To measure the prevalence and characteristics of distress and hope for the future among psycho-oncologists, who faced the coronavirus disease 2019 (COVID-19) emergency along with other healthcare workers.

**Methods:**

A web-based study was conducted among members of the Italian Society of Psycho-Oncology between May 29 and June 5, 2020.

**Results:**

A total of 237 members, aged 28–72 years, completed the COVID-19 Peritraumatic Distress Index (CPDI), Impact of Event Scale–Revised (IES-R), and HOPE questionnaires; 86.92% were female, 58.65% worked in hospitals, 21.10% were exposed to COVID-19, 11.39% experienced peritraumatic distress, and 3.38% had posttraumatic stress disorder symptoms. Peritraumatic distress was associated with living alone (adjusted odds ratio [AOR] 3.05; 95% confidence interval [CI] 1.41–8.13), using sleep remedies (AOR 3.79; 95% CI 1.41–10.21), and the perception of being avoided by family or friends because of work (AOR 2.69; 95% CI 1.02–7.11); high HOPE-Agency scores were associated with the absence of peritraumatic stress (AOR 0.40; 95% CI 0.16–0.96) after adjustment for age and sex.

**Conclusions:**

Psycho-oncologists showed greater resilience than other healthcare workers as they are trained to help others, but also to review their own values and behavior in light of stressful events. Of interest is the association between peritraumatic distress and social isolation, real or perceived. Healthcare institutions should pay attention to the mental well-being of their employees by promoting distress screening using simple tools such as the CPDI and implementing support interventions. Psycho-oncology associations should introduce policies aimed at developing a sense of social connectedness by providing an interactive system of orientation and scientific reference.

## Introduction

The coronavirus disease 2019 (COVID-19) pandemic constituted an unprecedented global crisis, an extreme public, economic, and social health emergency that changed everyone’s lives suddenly in an unimaginable way.^1^ The presence of an unknown virus, its rapid spread, and the lack of available treatment prompted about 215 countries, including Italy, to adopt individual and community containment measures. About a third of the world’s population was locked down in “the largest psychological experiment ever conducted.“^2^ In Italy, on June 1, 2020, the total number of cases diagnosed was 233,607; deceased 32,235; and healthcare workers affected 28,153.^3^

The population paid a high price with significant percentages of anxiety disorders, depression, sleep disturbances, and distress. In a recent meta-analysis on COVID-19’s psychological impact worldwide, the prevalence of anxiety and depression was 33% and 28%, even higher among people with preexisting health issues and COVID-19; there were no significant differences between healthcare professionals and the general population, except in countries with large numbers of cases, such as China, Italy, Turkey, Iran, and Spain.^4,5^

Lockdown and social distancing, although needed to flatten the case curve and/or keep the daily number of cases within hospital management capacity, contributed to economic as well as what has been described as a “social recession,” or an “epidemic of loneliness,”^6^ particularly critical for the more vulnerable, such as the elderly or people with disabilities.^7^

The pandemic profoundly changed clinical practice,^8^ but also affected the mental state and psychological conditions of health professionals at personal, family, social, and professional levels.^9^ The disruption of routine clinical practice, a feeling of loss of control, the fear of being a virus carrier, a sense of stigmatization, seeing patients in serious conditions and dying, uncertainty about pandemic developments, lack of adequate knowledge, and an overwhelming workload in a stressful environment with a high virus transmission rate were some of the factors causing psychic and physical symptoms,^10^ despite the presence of a high level of personal gratification for the work done.^11^

Despite uncertainties and inadequate preparedness—institutional and individual—and insufficient supply of protective devices, the national health service did what it could to manage the tsunami of serious cases, whose numbers exceeded the coping ability of intensive care units.^12^

All of this affected the mental health of healthcare workers.^4,9,13^ Psychologists continued to work during the pandemic emergency, both inside and outside hospitals, close up and at a distance, providing support for patients and healthcare workers. However, to our knowledge, their mental health during COVID-19 has been studied little or not at all. This may be because they are considered trained to be more resilient and less at risk of emotional distress. Nonetheless, existing literature has revealed burnout among clinical psychologists, with moderate or high levels of emotional exhaustion in 34%–50% of cases.^14,15^

Psycho-oncology, a discipline that lies among psychology, psychiatry, and oncology, is particularly active in Italy, with over 300 psycho-oncology services registered in hospitals or palliative care facilities. The Italian Society of Psycho-Oncology (SIPO), founded in 1985, is a scientific association of professionals (clinical psychologists, oncologists, psychiatrists, and other health professionals) who work in oncology-related sectors with patients with cancer, their families, and the multidisciplinary teams involved in cancer care, evaluating and treating the psychosocial consequences (www.siponazionale.it).

In the first COVID-19 pandemic wave (20 February–4 May 2020), defined by the Italian Ministry of Health and the Istituto Superiore di Sanità, acute and postacute cancer units had to reschedule their medical and surgical treatments and postpone follow-ups, screenings, and nonurgent examinations (http://www.trovanorme.salute.gov.it/norme/renderNormsanPdf?anno=2020&codLeg=73635&parte=1%20&serie=null). Psycho-oncologists worked in hospital wards and local communities, activating 140 help lines across the country. Face-to-face work consisted mainly of consultancy for hospitalized onco-hematologic patients and outpatients, even if some psycho-oncologists were assigned to COVID-19 wards. The latter involved the risk of contagion, since many Italian hospitals became “mixed” COVID-19 hospitals, with most beds assigned to patients with COVID-19. Indeed, cases of contagion among patients and staff often occurred.^9^ Remote work took place both in hospitals and from home through working and interviews via telephone help lines or telematic platforms.

The present study was drawn up by SIPO in response to recommendations issued by the World Health Organisation^16^ and the Inter-Agency Standing Committee^17^ in order to evaluate and monitor the psychological well-being of its members in relation to the COVID-19 emergency, also considering the greater risk of morbidity and mortality in the cancer population.

## Materials and methods

A descriptive-analytic, cross-sectional web-based study was conducted. The SIPO Institutional Review Board provided approval. Active members in good standing with active email addresses (n=394) were sent an anonymous questionnaire and asked to respond from May 29 to June 5, 2020, during the so-called epidemic transition phase that followed the first Italian pandemic wave. A total of 237 (60.15%) signed the online informed consent and completed the questionnaires.

All procedures followed in this study were in accordance with the World Medical Association 1964 Helsinki Declaration and its subsequent amendments.

### Instruments

The COVID-19 Peritraumatic Distress Index (CPDI),^18,19^ a 24-item questionnaire, refers to anxiety, depression, specific phobias, cognitive change, avoidance and compulsive behavior, physical symptoms, and loss of social functioning in the previous week. Each item is rated on a 5-point scale (from “not at all” to “extremely”). A score below 28 indicates no distress, between 28 and 51 mild to moderate distress, and above 51 severe distress. CPDI scores above 27 identify those operationally defined as peritraumatic stress cases. In this study, internal consistency was excellent, with Cronbach alpha coefficient = 0.833 (α = 0.934 and α = 0.871, for male and female respondents, respectively).

Impact of Event Scale–Revised (IES-R)^20^ is a 22-item questionnaire exploring the presence of probable posttraumatic stress disorder (PTSD). Feelings of distress during the previous week caused by “difficulties” listed are rated on a 5-point scale (from “not at all” to “extremely”), resulting in four scores: a total score and three separate scores measuring avoidance, intrusions, and hyperarousal. As with previous research, a cutoff = 50 was chosen to identify subjects with probable PTSD. Internal consistency was excellent to good for all scores, with Cronbach alpha coefficients ranging from 0.800 to 0.938.

HOPE^21,22^ is a 12-item questionnaire developed to measure hope in respect of the theory of Snyder et al.,^21^ modulated on two specific domains: agency indicates the mental capacity to plan the goals to be achieved and pathway refers to personal motivation in pursuing the set goals. Both components are necessary to increase the sense of hope. Four items measure agency (e.g. “I energetically pursue my goals”) and four measure pathways (e.g. “I can think of many ways to get out of a jam”); the remaining four are fillers. All are rated on an 8-point scale (from “definitely false” to “definitely true”) giving three scores: a total HOPE score and two separate scores that measure agency and pathways independently. The total HOPE score can range from 8 to 64; agency and pathway scores range from 4 to 32. Higher scores represent higher hope levels. Cronbach alpha coefficients ranged from 0.666 to 0.759.

Sociodemographic data (e.g. sex, age), workplace (e.g. at home, in a health unit, in a COVID-19 unit), lifestyle (e.g. type of home, religiosity), and COVID-19 exposure history (e.g. exposure, positivity, quarantine, hospitalization) were also collected.

Five questions indirectly concerning psychological aspects were asked at the end of the questionnaire (“Were you worried about dying if you contracted COVID-19?”; “Have you ever perceived that family and/or friends have avoided contact with you because of your work?”; “Have you received psychological support?”; “Have you used psychotropic drugs?”; “Have you used sleep remedies [drugs, supplements, herbal teas]?”).

### Data reduction and statistical analysis

To remove some variability in outcome at all covariate values while maintaining structure of the relationship between outcome and independent variables, the independent variables were categorized. Subjects were subdivided into two groups with respect to age (<51/⩾51 years),^19^ religious beliefs (no/yes), and geographic area of hospital work (Northern Italy/other geographic areas). In the absence of HOPE scale screening score studies, a median split on the two HOPE strategies were taken to create two groups (low vs high)—HOPE-Agency (<28/>27), HOPE-Pathways (<26/>25)—with the advantage of creating two equally numerous groups without being affected by extreme values. A variable called “psychological burden” was created as a sum of the “yes” responses to 7 items: exposure to COVID-19, positivity to COVID-19 test, concern about dying in case of contagion, avoidance by family and/or friends because of job, psychological support received, use of psychotropic drugs, use of sleep remedies.

The descriptive statistics included percentages or mean values, depending on the nature of each variable, as well as SDs whenever applicable. The correlations were estimated by computing Pearson coefficient *r*. Cronbach alpha was used to assess the internal consistency of scales.

Crude and adjusted odds ratios (AORs) with 95% confidence intervals (CIs) were calculated to study the association between selected variables and the propensity for peritraumatic stress (CPDI >27). Model-building involved assessing bivariate associations between the dependent variable and each of the potential covariates; covariates not significantly associated (*p* > 0.10) with the outcome were then excluded from further consideration. The remaining candidate covariates underwent multivariable logistic regression and were subjected to backward selection until all remaining covariates had *p* value <0.05. Age and sex were treated as confounding variables.

No questionnaire was excluded from analysis for missing values. Analyses were conducted on a sample of 237 psycho-oncologists.

All statistical analyses were performed using STATA, version 11.0 (StataCorp, College Station, TX), with statistical significance set at *p* = 0.05.

## Results

Results are given in Tables 1 and 2.

**Table 1.** Differences between COVID-19 Peritraumatic Distress Index results of no peritraumatic distress (n = 210) or peritraumatic distress (n = 27) on variables in study.

	No distress	Distress	*p* Value
Total	210 (88.61)	27 (11.39)	
Age, y	45.20 ± 10.28	46.41 ± 11.41	0.571
Live alone	30 (14.29)	9 (33.33)	0.012
Work			
At home	22 (10.48)	3 (11.11)	
Out of home	46 (21.90)	1 (3.70)	
Hospital	123 (58.57)	16 (59.26)	
Other	19 (9.05)	7 (25.93)	0.017
Exposed to COVID-19	46 (21.90)	4 (14.81)	0.395
COVID-19 positive	6 (2.86)	1 (3.70)	0.446
Worried about dying	6 (2.86)	0	0.374
Avoided by others	28 (13.33)	8 (29.63)	0.026
Psychological support	31 (14.76)	8 (29.63)	0.050
Psychotropic drugs	6 (2.86)	3 (11.11)	0.035
Sleeping remedies	22 (10.48)	9 (33.33)	0.001
IES-R Intrusion	3.37 ± 3.83	12.26 ± 5.33	<0.001
IES-R Avoidance	3.87 ± 3.76	10.93 ± 4.23	<0.001
IES-R Hyperarousal	3.187 ± 3.00	11.44 ± 4.52	<0.001
IES-R Total	10.42 ± 9.44	34.63 ± 11.76	<0.001
HOPE-Agency	27.37 ± 2.59	25.78 ± 4.22	0.006
HOPE-Pathways	24.43 ± 4.38	24.04 ± 4.97	0.667
HOPE-Total	51.80 ± 6.04	49.81 ± 8.88	0.132

COVID-19: coronavirus disease 2019; IES-R: Impact of Event Scale–Revised.

Values are n (%) or mean ± SD.

**Table 2.** Univariate and multivariable logistic regression analysis.

	N	%	OR	95% CI	AOR^a^	95% CI
Sex						
Male	29	12.24	Reference		Reference	
Female	206	86.92	0.75	0.24–2.35	0.58	0.16–2.02
Other	2	0.84	(Omitted)		(Omitted)	
Age, y						
<51	166	70.04			Reference	
>50	71	29.96	1.19	0.51–2.80	0.92	0.35–2.43
Live alone						
No	198	83.54	Reference		Reference	
Yes	39	16.46	3.00	1.23–7.30	3.05	1.14–8.13
Cohabitants						
0	39	16.46	Reference			
1	58	24.47	0.46	0.15–1.36		
2	51	21.52	0.36	0.11–1.19		
3–5	89	37.55	0.24	0.08–0.73		
Work						
At home	25	10.55	Reference			
Out of home	47	19.83	0.16	0.22–1.62		
Hospital	139	58.65	0.95	0.26–3.55		
Other	26	10.97	2.70	0.61–11.93		
Work in hospital: northern Italy						
No	164	69.20	Reference			
Yes	73	30.80	0.61	0.24–1.58		
Residence area in Italy						
North	101	43.53	Reference			
Center	45	19.40	1.28	0.40–4.05		
South and isles	86	37.07	1.82	0.74–4.49		
Exposure to COVID-19						
No	187	78.90	Reference			
Yes	50	21.10	0.62	0.20–1.88		
Positive to COVID-19						
No	181	76.37	Reference			
Yes	7	2.95	1.51	0.17–13.25		
No swab	49	20.68	1.77	0.72–4.35		
Worried about dying						
No	231	97.47				
Yes	6	2.53	(Omitted)			
Avoided by others						
No	201	84.81	Reference		Reference	
Yes	36	15.19	2.74	1.09–6.85	2.69	1.02–7.11
Psychological support						
No	198	83.54	Reference			
Yes	39	16.46	2.43	0.98–6.04		
Psychotropic drugs						
No	228	96.20	Reference			
Yes	9	3.80	4.25	1.00–18.10		
Sleeping remedies						
No	206	86.92	Reference		Reference	
Yes	31	13.08	4.27	1.71–10.66	3.79	1.41–10.21
IES-R (>50)						
No	229	96.62	Reference			
Yes	8	8.38	29.71	5.74–156.59		
HOPE-Agency (>median)^b^						
No	104	43.88	Reference		Reference	
Yes	133	56.12	0.42	0.18–0.95	0.40	0.16–0.96
HOPE-Pathways (>median)^b^						
No	129	54.43	Reference			
Yes	108	45.57	0.95	0.42–2.13		
Psychological burden^c^						
No	117	49.37	Reference			
Yes	120	50.63	3.92	1.52–10.11		

AOR: adjusted odds ratio; CI: confidence interval; COVID-19: coronavirus disease 2019; IES-R: Impact of Event Scale–Revised; OR: odds ratio.

COVID-19 Peritraumatic Distress Index cases of peritraumatic stress disorder as dependent variable.

a There were no missing values. Two were excluded from multivariate logistic regression for sex value equal “other.”

b Score above the sample median.

c Sum of “yes” responses to 7 items: exposure to COVID-19, positivity to test for COVID-19, worried about dying in the case of contagion with COVID-19, avoided by family and/or friends because of work done, receive psychological support, use psychotropic drugs, use remedies to sleep.

In Table 1, the descriptive statistics are divided into those with CPDI scores lower than 28 (no distress, n=210) and those with CPDI scores higher than 27 (peritraumatic distress, n = 27).

There was a significant association between the area of residence (north, center, south, and islands) and exposure to COVID-19 and positivity to the test, with the latter significantly higher among respondents working in northern Italy than in other Italian areas (52.90%, 17.39% and 29.71%, vs 29.79%, 22.34%, and 47.87%; *p* = 0.002, *p* = 0.001).

There were significant correlations between CPDI and IES-R scores (intrusion, *r* = 0.69; avoidance, *r* = 0.65; hyperarousal, *r* = 0.81; total IES-R, *r* = 0.78). These values were higher for men (intrusion, *r* = 0.82; avoidance, *r* = 0.75; hyperarousal, *r* = 0.94) than for women (intrusion, *r* = 0.67; avoidance, *r* = 0.63; hyperarousal, *r* = 0.79). The correlation coefficients between CPDI and HOPE-Agency or HOPE-Pathways scores were very low (HOPE-A, *r* = −0.33; HOPE-P, *r* = −0.16; total HOPE score, *r* = −0.25).

Twenty-seven (11.39%) were cases of peritraumatic distress and 8 (3.38%) PTSD. About half had psychological burden.

In univariate logistic regression analyses, a number of variables were significantly associated positively with peritraumatic distress: living alone, receiving psychological support, using psychotropic drugs, using sleep remedies, and perceiving being avoided by family and friends for the work performed. Living with 3 or more others was significantly associated negatively.

At logistic regression, multivariable analysis adjusted for confounding factors use of sleep remedies (AOR 3.79; 95% CI, 1.41–10.21), living alone (AOR 3.05; 95% CI, 1.41–8.13), the perception of being avoided by family and/or friends because of one’s job (AOR 2.69; 95% CI 1.02–7.11), and HOPE-Agency (AOR 0.40; 95% CI 0.16–0.96) were significant, independent factors associated with peritraumatic distress.

## Discussion

The Italian government declared a state of emergency on January 31, 2020, and after the WHO officially announced the COVID-19 pandemic ordered a complete lockdown of the country on March 11. This continued until May 4, when the epidemic transition phase began with a gradual and progressive return to commercial and productive activities.

The first interesting result of our survey is that the prevalence of peritraumatic distress among psycho-oncologists was 11.39%, significantly lower than that reported for other healthcare workers (21.9%–71.5%)^9^ and the general population (30.9%–35.0%).^18,19,23^ The symptoms of PTSD, as measured by the IES-R, were present in 3.38%, compared to 49.4% reported by a recent Italian study on healthcare workers.^24^ Nevertheless, when looking at the indicators of psychological burden mentioned previously, about half the psycho-oncologists in the sample were found to have it. However, most of them showed elevated levels of HOPE-Agency and HOPE-Pathways, thus showing a positive motivational state and a vision for the future.

It does not seem surprising that psycho-oncologists showed greater resilience than other healthcare workers and not just because they were not on the front lines. As mental health professionals in oncology and palliative care, they are trained to help patients, their families, and members of therapeutic teams, but also to deal with the limits imposed by cancer and the sharp existential fractures it entails, to review their values in the light of changes, and to reschedule their lives from a new perspective.

The percentage of those afraid of dying if infected was very low compared to the general population (2.53% vs 27.36%).^19^ The dimension of death and dying, the ability to tolerate its resonances, to process mourning even indirectly through the patients’ events, is an integral part of their daily work. The emotional and personal preparation in adapting to stressful situations is important and the personal training of a psycho-oncologist is also based on the acquisition of the ability to re-elaborate events and manage stress. It is possible that hope for the future shown by the high scores on the HOPE scale acted as a protective factor cushioning the impact of the pandemic. How we perceive the future can greatly affect how we feel in the present. Hope theory suggests that hope in the future is related to positive physical and mental health outcomes and provides a psychological resource that can help individuals respond to trauma with resilience.^25^ Work on hope for the future is a central theme in oncology and the ability to maintain and reprogram hope despite a serious prognosis or at the end of life is the fulcrum of many existential psycho-oncologist interventions.^26,27^ Previous studies have shown that HOPE-Agency and HOPE-Pathways are negatively correlated with depression and PTSD.^25^

Nevertheless, there were some cases of moderate to severe peritraumatic distress. PTSD was associated with social isolation, whether real or perceived (feelings of being avoided by relatives and friends because of work), living alone, and taking sleep remedies. High goal-directed energy, as measured by HOPE-Agency, was a protective factor.

Isolation and loneliness are problems that affect a large part of the general population. In the United States, 35% of adults over 45 and 43% of adults over 60 feel lonely.^7,28^ The feeling of loneliness is usually amplified by the loss of a loved one, chronic disease, or loss of sight or hearing, but it can also be linked to working conditions.^29^

The obligation of social distancing in many countries for the purpose of containing COVID-19 increased social isolation (objective scarcity of social connections) and loneliness (subjective perception of feeling alone). Over 3 billion people worldwide were confined to their homes. In Italy, a tight lockdown prohibited people from leaving their homes if not for urgent reasons and closed down churches, parks, restaurants, bars, and other commercial activities from March 11 to May 4, 2020. This caused an epidemic of loneliness, a social recession,^6^ an interruption of people’s everyday lifestyle, as well as feelings of fear associated with a situation considered out of control.^30^

A recent article on the cruelty of changes in our lives stated “We are starved for physical contact with our loved ones.”^31^ Humans have evolved to feel safe in a group and experience isolation as a physical state of emergency.^32^ Social isolation and loneliness are not only social problems, but affect physical and mental health and are known as major risk factors for morbidity and mortality.^33,34^ We know that loneliness is a psychological state, a stressful experience that derives from deficits in a person’s social relationships, both qualitative and quantitative,^35^ caused by individual personality traits but also by social situations and environmental characteristics.

When the environment does not provide adequate conditions, even a well-adjusted person can develop behaviors and thoughts typically attributed to individuals who feel alone.^36^ Contacts, and physical contact, are necessary for a condition of well-being.^37^ This is confirmed by recent studies that identify increased peripheral oxytocin as a marker of social and interpersonal distress and a signal of the need to seek social affiliation.^38^ In a recent study on 180 medical workers who treated patients with COVID-19, social support was positively and significantly associated with self-efficacy and sleep quality and negatively associated with the degree of anxiety and stress.^39^ In our study, psycho-oncologists who lived alone or who perceived stigmatization were more affected than others, showing higher levels of distress.

In Italy, the closure of psycho-oncology clinics resulted in most psycho-oncologists working, often alone, in hospitals using telephone help lines/telematic platforms. The difficulty in sharing their feelings of fear and sadness for the infected and the dead, their concern for the reconversion of entire hospitals, their empathy with hospitalized patients with cancer alone in the wards due to the ban on visitors, as well as the lack of group interactions and face-to-face relationships that feed what has been called protective “relational energy”^40^ surely affected the quality of their relationships in psychological interventions.

As one doctor said, “I am rarely able to get to the heart of the matter by phone.”^41^ Another objected, “I find myself wholly unprepared to speak of death and dying across cell phones or video links with unreliable connections. I have not yet figured out how to help guide patients’ struggles with cancer—leading them toward a death with dignity and finding personal reward in our relationship—when I cannot see them, hug them, or see their love for each other. What does it take to find balance and connection in virtual oncology?”^42^

Feeling vulnerable to contagion in the workplace, and using personal protective equipment that limits nonverbal communication, may also have weighed on the sense of isolation and loneliness.

### Study limitations

There are some limitations to this study. The first is the number of respondents: 237 out of 394 (60.15%). Questionnaires were sent to all SIPO members with the hope that most of them would reply. This was not the case, but we have no way of knowing the characteristics of those who did not reply (age, sex, etc.) as the questionnaires were anonymous: those who did not reply may have had PTSD, been ashamed to declare being in a state perceived as inadequate, had a very demanding workload, or refused to respond to yet another online survey. We nevertheless are confident that our sample represents the whole group well because the geographic distribution is coherent with the members’ regions of residence, as well as the percentage of psycho-oncologists who work in hospitals.

A second limitation is the use of questionnaires. To guarantee greater adhesion, we chose to use a limited number that were easy to administer. However, as screening tools they do not allow us to speculate on the psychological health of the respondents. In addition, psychologists have a great deal of experience with questionnaires and may have responded less sincerely (and in a way that is more socially desirable). Despite this, the data on real or perceived social isolation and psychological burden emerged clearly.

## Conclusions

Stress reactions, anxiety, insomnia, fear, anger, and bewilderment are normal responses to events such as pandemics that threaten the life and survival of individuals and the entire human community. Overall, the psycho-oncologists in our sample seem to have adapted successfully and showed resilience and the ability to maintain a positive vision of the future more than other healthcare professionals. However, a subgroup proved more vulnerable, reporting symptoms of peritraumatic distress associated with conditions of greater isolation and loneliness that exacerbated concomitant or preexisting factors.

“We don’t have a word for the opposite of loneliness, but if we did, I could say that’s what I want in life,” wrote Marina Keegan in a piece in *Yale News* at the end of her university studies, sad to have to leave that sense of connection, community, togetherness, and abundance of people she had been close to in previous years.^43^ A Stanford psychiatrist, paraphrasing Keegan, wonders, “Is there a word for the absence of an embrace during a pandemic? Does the phrase social distancing capture what it means to be kept apart?”; emphasizing the difficulty in describing in a word the combination of our tendency to unite and the sense of duty to remain separate, the fear of becoming infected, and the sense of emptiness that that empty space leaves us.^37^

Psycho-oncologists do hard work: they are sponges that empathically filter the physical, psychological, social, and spiritual pain of losses related to cancer, and help transform them into tolerable experiences and thoughts. Thus, they need a psychic recharge from various sources such as their continuous training, co-vision work with colleagues, but also meaningful emotional relationships outside of work, a healthy sex life, hobbies that enlighten the mind, social relationships, and other possible sources of pleasure—elements that may be diminished or missing in a lockdown.

Healthcare institutions should be alert to the mental well-being of their staff, promoting distress screening programs using simple assessment tools such as the CPDI and implementing support interventions for those who have difficulty in adapting. Psycho-oncology companies should introduce corporate policies aimed at developing a sense of social connectedness by functioning as an active and interactive system of orientation and scientific reference. Pandemic programs communicating that challenges have to be faced together^44^ should sensitize their members to factors that promote pandemic resilience: training/updating centered on those areas of emotional intelligence such as self-awareness and self-management considered relevant for mental health, especially in times of crisis.^45^
